# Conformational and structural stability of *n* and 2-propylthiols: a revisit†

**DOI:** 10.1039/d2ra01034h

**Published:** 2022-04-01

**Authors:** Manish Kumar Tripathi, Venkatnarayan Ramanathan

**Affiliations:** Department of Chemistry, IIT (BHU) Varanasi UP India vraman.chy@iitbhu.ac.in

## Abstract

The conformational and structural stability of *n*-propanethiol (nP) is revisited owing to the prevailing ambiguity in the literature reported hitherto, and the rationale for 2-propanethiol's (2P) most stable conformers is analyzed. Based on the rotation around the C–C and C–S bonds, four conformers for nP and two conformers for 2-propanethiol (2P) were found to have the lowest energies at the CCSD/cc-pVDZ level of theory. The two conformers of 2P are anti (T), and gauche (G), and those of nP are T–G, G–G, T–T, and G–T. Rotational barriers, geometrical parameters, fundamental vibrational modes, and energy parameters reported herein agree exceedingly well with the reported experimental values for nP and 2P molecules. Furthermore, natural bond orbital (NBO), frontier molecular orbital (FMO), Mulliken charge (MC), electrostatic potential charge (ESP), and vibrational mode analyses were carried out to get a better understanding of both the thiols.

## Abstract 2

C–C and C–S bond rotations give four minima for nP molecules. Computational predictions reinforced by ESPM, MCA, NBO, FMO and NCI analyses excellently matched with experimental results.

## Graphical abstract

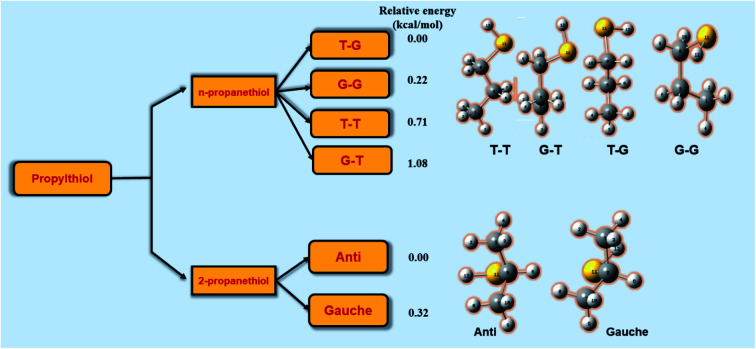

## Introduction

*n*-Propanethiol (nP) and 2-propanethiol (2P) are molecules of importance from astrochemistry.^1^ Precise knowledge of the lowest energy conformers is of paramount significance. Corresponding to the rotation around the C–C and C–S bonds, nP can take up seven possible conformers, namely trans–trans (T–T), trans–gauche (T–G), gauche–trans (G–T), gauche–gauche (G–G), gauche–gauche (G–G′), trans–gauche (T–G′), and gauche–trans (G′–T); and 2P has two confomers namely, anti (T) and gauche (G). In experiments, not all these conformers are found, though. Different researchers reported varying numbers and structures of conformers using different spectroscopic techniques like microwave,^2–5^ infrared,^6,7^ Raman,^8^ and vacuum ultraviolet mass-analyzed threshold ionization spectroscopy.^9^ On the other hand, literature pertaining to computational research using methods such as Urey–Bradley force field theory,^10^ quantum molecular force field theory,^11^ MP2/cc-pVTZ, and B3LYP/6-311++G(2df, 2pd)^9^ differed significantly among each other as well as from the experiments.

Whereas the infrared absorption-based experiments detected the conformers T–T and G–T^6^ as the global and local minima, the microwave-based rotational spectroscopic studies^3^ reported that T–G and T–T were the global and local minimum. The matrix-assisted threshold ionization spectroscopy reported by Choi *et al.* also found two conformers, T–G and G–G.^9^ Based on their computational predictions using Franck–Condon calculations, they identified T–G as the global minimum and G–G as the other low-lying conformer amongst the five conformers (T–G < G–G < G–G′ < T–T < G–T) using B3LYP/6-311++G(2df, 2pd) level of theory.^9^ The most recent work in this regard was done by Gorai *et al.*,^1^ who predicted the same order of conformers' energy as T–G < G–G < G–G′ < T–T < G–T using B3LYP/6-311++G(2df, 2pd) level of theory. Although Choi *et al.*^9^ and Gorai *et al.*^1^ employed an identical level of theory, their computational results vary significantly although the global minimum identified by both is T–G, as mentioned above.

It is noteworthy to say that the predictions shown in this report match extremely well with those of experimental predictions.^3,6^ Despite Choi *et al.*'s disparity in identifying the correct local minima conformers, they ascribe the erroneous conformer to the experimentally observed one based on Franck–Condon calculations. The most recent predictions by Gorai *et al.* are also farther from experimental results.

Although Hayashi *et al.* reported T–G as the global minimum conformer of nP by carrying out the rotationally resolved spectroscopy^3^ and subsequent works based on other experiments relied on the computational predictions in ascribing the correct conformational information to the structures detected in their experiments, furthermore, in the case of 2P, the value corresponding to the rotational barrier needs revision as the latest theoretical work^11^ cites a relatively older experimental observation^12^ completely ignoring the latest experimental value^13^ for no understandable reasons. Hence the accuracy and correctness of the computational prediction are of paramount importance as they go beyond merely supporting the experimental findings. Furthermore, there is no consensus on the correct sequence of the energies and the number of other low energy conformers. In other words, the rationale of using the computational predictions to support the experimental findings is not completely satisfactory.

In order to fill the gaps pertaining to the low energy conformations of nP and 2P molecules, we re-investigated the conformational and structural stability of the propylthiol molecule using state of the art computational methods. The results reported herein correlate exceedingly well with the available experimental results for the propylthiol molecular system.^1–3,6,7,9,10^ Computations of normal modes and electrostatic potential charge (ESP), Mulliken charge analysis (MCA), natural bond orbital analysis (NBO), frontier molecular orbital (FMO), and non-covalent interaction (NCI) were also carried out to support further the results pertaining to the conformational stability of nP and 2P molecules. It is envisaged that with the results reported herein, the unambiguity pertaining to the conformational aspects of nP would cease, and these results may serve as a benchmark for conformational analysis of the propylthiol molecular system.

## Computational method

**Fig. 1:**
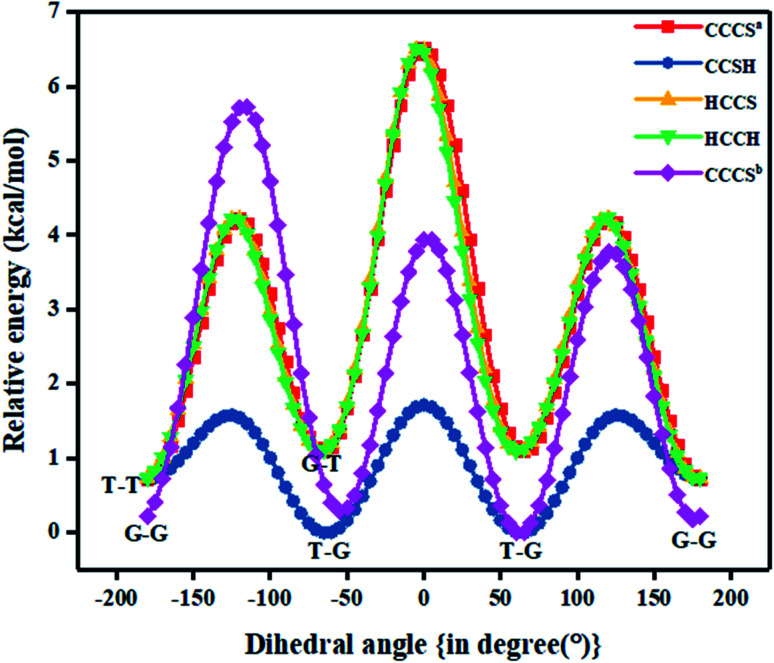
Various relative potential energy curves obtained at CCSD/cc-pVDZ level of theory with respect to T–G conformer (clockwise relaxed scan with step size 5°) for nP molecule (in image term ‘a’ and ‘b’ represent optimized geometry of T–T and G–G conformer that was taken into consideration).

Geometry optimizations, normal mode calculations, and natural bond orbital analysis (NBO) for all conformers of nP and 2P were carried out using the CCSD/cc-pVDZ level of theory. Potential energy surface was also generated at the CCSD/cc-pVDZ level of theory with step size 5° (containing 73 points) variation in all the dihedral angles such as 1–5–8–11, 5–8–11–12, 6–5–8–11, and 6–5–8–9 [atom numbering as per Fig. 1]. A potential energy surface was also generated at the B3LYP/6-311++G(2df, 2pd) level of theory by varying the dihedral angles 1–5–8–11, and 5–8–11–12 with a step size of 10° and 5° (refer Fig. SI4†). A 2-dimensional potential energy surface was generated by simultaneously varying two dihedral angles, namely 1–5–8–11 and 5–8–11–12 with step size 30° (containing 144 points) at CCSD/cc-pVDZ level of theory. Single point calculations were performed at HF, MP2, MP3, MP4, CCSD, and CCSD(T) levels of theory using cc-pVNZ (where N= D, T, Q) basis sets for all conformers of nP. Helgaker method was utilized to extrapolate correlation energy, and results were obtained with basis sets cc-pVNZ (where N = T, Q).^14^

1 *E* = *a* + *bX*^−3^

where *X* is the cardinal number *i.e.*, four for quadruple-zeta sets and three for triple-zeta settings *etc.*

Population of different conformers was calculated using the Boltzmann distribution equation:^15^2
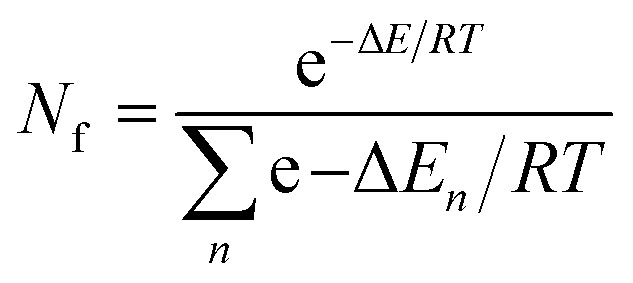
where Δ*E* represents the difference in energy of conformer with respect to the global minima conformer and summation applied over all *n* possible conformers, *R* = 1.987 × 10^−3^ kcal K^−1^ mol^−1^ and *T* = 298 K.

### NBO analysis

For each donor, NBO(*i*) and acceptor NBO(*j*), the stabilization energy *E*(2) associated with the delocalization *i*  →  *j* are given by the following equation^15^3
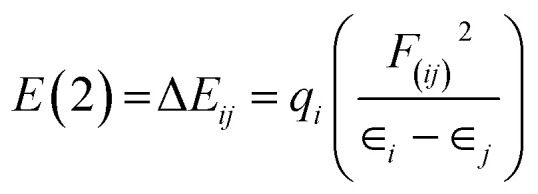
where *q*_*i*_ is the *i*^th^ donor orbital occupancy, ∈_*i*_ and ∈_*j*_ are diagonal elements (orbital energies), and *F*_(*ij*)_ is the off-diagonal NBO Fock matrix elements.

### FMO analysis

To derive energy parameters from the FMO analysis, we utilize results of the FMO analysis for accreditation of the global reactivity terms such as chemical potential (*μ*), hardness (*η*), electrophilicity index (*ω*), and electronegativity (χ) that are derived from the following equations.^15^4
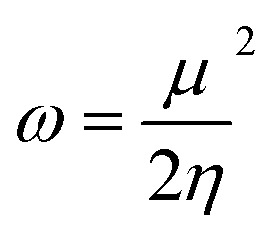
5
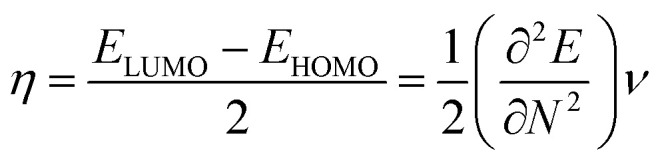
6
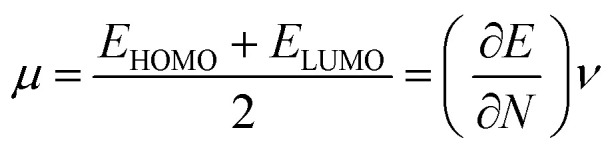

7 *χ* = −*μ*

Corresponding to low energy conformers, Mulliken charge analysis (MCA), electrostatic potential charge (ESP), non-covalent interaction (NCI) representation, natural bond orbital (NBO) analysis, and frontier molecular orbital (FMO) analysis were performed at the CCSD/cc-pVDZ level of theory. MultiWFN software was used for NCI calculations,^16^ and Gaussian 16 (ref. 17) was utilized for all other calculations.

## Result and discussion

### Conformational analysis

**Fig. 2:**
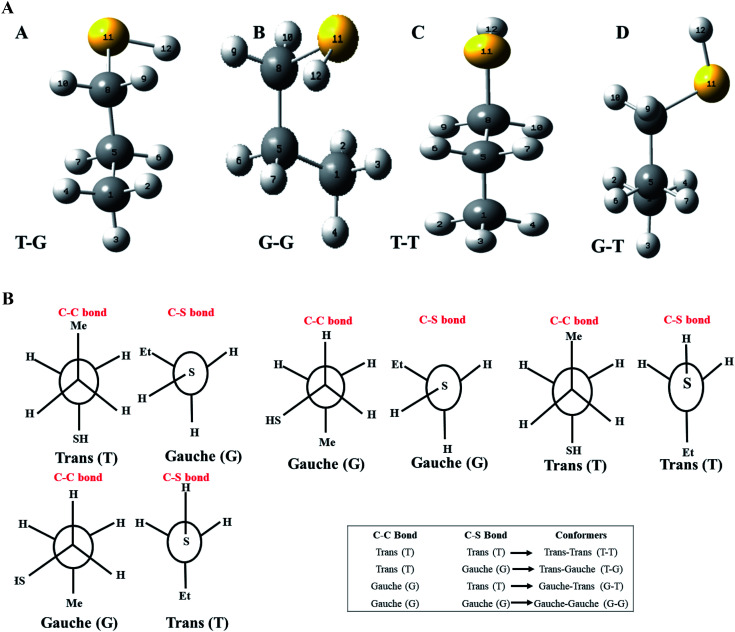
(a) Optimized geometries of different conformers of nP at CCSD/cc-pVDZ level of theory (configuration of conformers T–T, G–T, and T–G are given correspond C–C and C–S bonds respectively and here T represents trans and G represents gauche conformation). (b) Newman projection for conformers of nP molecule with respect to C–C and C–S bonds.

Optimized geometries of the low energy conformers of nP and 2P molecules are shown in Fig. 2(a) and SI1.† All these geometries were bereft of any imaginary frequency (Table ST1 in ESI† summarizes normal mode analysis). The geometric parameters, whose experimental values are available, are summarized in Table 1 for nP and 2P molecules, and the same computed at CCSD/cc-pVDZ support the experimental results very well, which have hitherto not been reported in the computational literature. Taking cue from this, the CCSD/cc-pVDZ has been used further for the remaining calculations for both nP and 2P molecules.

Geometrical parameters and rotational barriers of the different conformers of the nP & 2P molecules at CCSD/cc-pVDZ level of theory [symbols *R*, *A*, and *D* represents the bond distance (angstrom (Å)), bond angle (°), and dihedral angle (°) respectively]^a^

**Table:** 

Geometrical parameters (*n*-propanethiol)						
Parameter	T–T	Exp.^3^	T–G	Exp.^3^	G–G	G–T
*R* (1, 2)	1.106	1.094	1.106	1.094	1.107	1.106
*R* (1, 5)	1.534	1.536	1.535	1.536	1.533	1.535
*R* (8, 11)	1.841	1.814	1.837	1.820	1.840	1.845
*R* (11, 12)	1.352	1.336	1.353	1.336	1.353	1.352
*A* (2, 1, 5)	111° 30′	111° 0′	111° 48′	111° 0′	110° 41′	110°.45′
*A* (5, 8, 11)	109° 33′	108° 34′	114° 10′	113° 37′	114° 52′	110° 24′
*A* (8, 11, 12)	96° 27′	96° 13′	96° 10	96° 00′	95° 53′	96° 36′
*D* (5, 8, 11, 12)	−180° 00′	180° 00′	−63° 17′	61° 45′	−65° 15′	−176° 38′

a Here numbering patterns are as per Fig. 2 and 5, and the term ‘a’ represents geometrical parameters of anti-conformation correspond to C–S bond and the term ‘b’ represents rotational barrier at B3LYP/6-311++G (2df, 2pd) level of theory.

**Table:** 

Geometrical parameters (2-propanethiol)				Rotational energy barrier (C–S) at CCSD/cc-pVDZ level of theory			
Parameter	Gauche	Anti	Experimental	Our results		Experiment^3,16^ (kcal mol−^1^)	
				nP	2P	nP	2P
*R* (1, 2)	1.105	1.107	1.091^a^	1.58 (1.54)^b^	1.72	1.31 (ref. 3)	1.88 (ref. 16)
*R* (1, 5)	1.532	1.534	1.520^a^				
*R* (5, 11)	1.850	1.847	1.849^a^				
*R* (11, 12)	1.353	1.354	1.345^a^				
*A* (4, 1, 5)	111° 30′	111° 12′	113° 36′^a^				
*A* (1, 5, 11)	111° 48′	111° 48′	111° 12′^a^				
*A* (5, 11, 12)	96° 12′	95° 36′	96° 30′^a^				

Fig. 1 also shows the potential energy curve generated through a relaxed scan on rotating the C–C and C–S bonds of the nP molecule where the dihedral angle was varied in steps of 5°, and the optimized geometry of the T–T and G–G conformers were the starting geometry. Four such potential energy curves were obtained corresponding to dihedral angles 1, 5, 8, 11 (C–C–C–S); 5, 8, 11, 12 (C–C–S–H); 6, 5, 8, 11 (H–C–C–S), and 6, 5, 8, 9 (H–C–C–H). These scanning coordinates resulted in two conformers with minimum energy, as shown in Fig. 1 and SI2† and Table 1. Scanning coordinates 1, 5, 8, 11; 6, 5, 8, 11 and 6, 5, 8, 9 provide T–T and G–T conformers as a minimum, whereas T–G and T–T conformers were found corresponding to the dihedral angle 5, 8, 11, 12, which is similar to the one reported by Hayashi *et al.*^6^ and Nakagawa *et al.*^3^ respectively. Experimental measurements reported T–G as the global minimum (although one earlier experiment based on IR absorption identified T–T as the global minimum) with one local minimum conformer G–T or T–T conformers, respectively.^2–8^ The scan with initial geometry as the optimized G–G conformer resulted in T–G as the global minimum conformer and G–G as the local minimum which is similar to the prediction by Gorai *et al.*^1^ and Choi *et al.*^9^ Furthermore, the results reported herein match exceedingly well with the experimental results. Table ST2† summarizes all conformational scans for the nP molecule at the CCSD/cc-pVDZ level of theory with a step size of 5°.

The C–S rotational barrier of nP molecule is computed to be 1.58 kcal mol^−1^ and 2.24 kcal mol^−1^ from the corresponding scans with initial geometries T–T and G–G, respectively where the experimental value for the C–S rotational barrier is 1.31 kcal mol^−1^.

It was observed that when the scan was carried out by varying the CCSH dihedral angle, the C–C bond got significantly distorted (up to 13%), and the C–S bond got distorted up to 5.4%. It is evident that the C–C bond plays a crucial role in determining the low energy conformer along with the C–S bond. This reinforces the fact that along with the C–S bond, the C–C bond should be considered to render the conformational analysis of the nP molecule complete. Past research reports have only resorted to the C–S bond rotation.

**Fig. 3:**
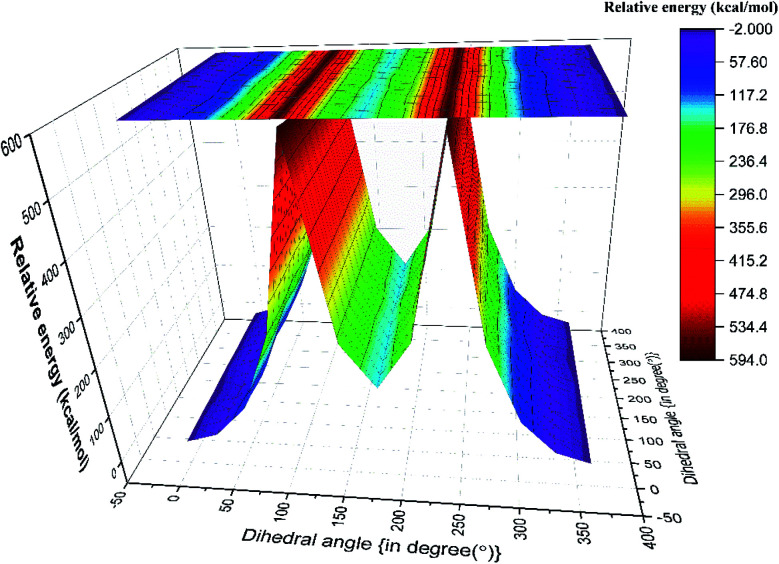
Potential energy surface plot generated for nP molecule with two coordinates (CCCS and CCSH) with step size 30° at CCSD/cc-pVDZ level of theory (geometry of T–T conformer as a starting molecule).

Although both C–C and C–S bonds were considered for the analysis reported herein, the analysis was carried out separately. Taking a cue from the individual analysis *i.e.*, the rotations distort the bonds, calculations were extended further (Fig. 3) where both the rotations were carried out simultaneously. The resulting potential energy 2D surface revealed T–T as the global minimum instead of the T–G conformer when we considered T–T optimized geometry as the starting configuration. With the optimized geometry of the G–G conformer as the initial point for the double scan varying the C–S and C–C bond simultaneously is shown in Fig. SI3.† The global minimum conformer obtained from the 2D scan of the T–T conformer and the global minimum got from the three C–C bond rotations (Fig. 1) were identical.

The computed value of the rotational barrier (Table ST2†) for the nP molecule corresponding to the three C–C bond rotations is 3.12 ± 0.2 kcal mol^−1^ which too matches well with the experimental value of 2.9 kcal mol^−1^.^18^

In Fig. 1 the T–G conformer is taken as the reference and the energies of other conformers are depicted with respect to this reference. In the studied models, the total energy of the nP molecule was calculated concerning dihedral angle, which is summarized in Tables 2 and ST3.† The energy difference between the global minimum (T–G) and the local minima (G–G), calculated at the CCSD(T)/CBS limit, was 0.30 kcal mol^−1^, which matched very well with the experimental value (0.38 kcal mol^−1^) as shown in Tables 2 and ST3.†^3^ However, the value of the energy difference turned out to be 0.22 kcal mol^−1^ when the geometries were optimized at the CCSD/cc-pVDZ level of theory. Apart from this, the relative energy of the other two conformers is 0.71 kcal mol^−1^ and 1.08 kcal mol^−1^ for T–T and G–T conformers at the CCSD/cc-pVDZ level of theory, respectively. The predicted energy difference was further verified by carrying out population analysis, summarized in Table 3. From Table 3, it is evident that when the entire conformational space is explored by varying the C_α_–S and the C_α_–C_β_ bonds, the conformers T–G and G–G seem to have the maximum populations followed by the T–T conformer. Earlier computational works concentrated only on the C_α_–S, which was adequate to understand the global minimum but predictions varied for the local minimum. Herein it is shown unambiguously that the C_α_–C_β_ should also be considered for the accurate prediction of the local minima conformer. Our results support all previous experimental measurements.^2–7^

**Table:** Relative change in single-point energy (kcal mol^−1^) of the local minima conformer of the nP (T–T) and 2P with respect to the global minimum conformer (T–G for nP) at different levels of theory^a^

Basis set→	CBS limit		Experiment		Optimized energy by Choi *et al.* @B3LYP/6-311++G(2df, 2pd)^8^	
Methods↓	nP	2P	nP^3^	2P^16^	nP	2P
HF	0.78	0.07	0.38	0.06	0.62 (TG–TT)	0.05
MP2	0.16	0.00				
MP3	0.30	0.03				
MP4	0.28	0.03			0.50 (TG–GG)	
CCSD	0.30	0.03				
CCSD(T)	0.22	0.03				

a Energy difference between local and global minimum conformer for the nP molecule is 0.61 kcal mol^−1^ at B3LYP/6-311++G (2df, 2pd) level of theory.

**Table:** Conformational abundances of the nP and 2P molecules at CCSD/cc-pVDZ level of theory^a^

Dihedral angle	*n*-Propanethiol										2-Propanethiol	
	1, 5, 8, 11^a^ (CCCS)		5, 8, 11, 12^a^ (CCSH)		6, 5, 8, 11^a^ (HCCS)		6, 5, 8, 9^a^ (HCCH)		1, 5, 8, 11^b^ (CCCS)		6, 5, 11, 12 HCSH	
	Δ*E* (kcal mol^−1^)	*N*_f_ (%)	Δ*E* (kcal mol^−1^)	*N*_f_ (%)	Δ*E* (kcal mol^−1^)	*N*_f_ (%)	Δ*E* (kcal mol^−1^)	*N*_f_ (%)	Δ*E* (kcal mol^−1^)	*N*_f_ (%)	Δ*E* (kcal mol^−1^)	*N*_f_ (%)
0	0.00	**33.8**	0.72	**10.7**	0.00	**33.4**	0.00	**33.2**	0.22	**23.0**	0.00	**30.1**
60	3.51	0.1	1.54	2.7	3.49	0.1	3.47	0.1	5.72	0.0	1.73	1.6
120	0.44	**16.1**	0.00	**35.7**	0.46	**15.3**	0.45	**15.5**	0.29	**20.4**	0.32	**17.5**
180	5.80	0.0	1.71	2.0	5.76	0	5.73	0.0	3.95	0.0	1.78	1.5
240	0.44	**16.1**	0.00	**35.7**	0.37	**17.8**	0.36	**17.9**	0	**33.3**	0.32	**17.5**
300	3.51	0.1	1.54	2.7	3.50	0.1	3.50	0.1	3.9	0.0	1.73	1.6
360	0.00	**33.8**	0.72	**10.7**	0.00	**33.4**	0.00	**33.2**	0.21	**23.0**	0.00	**30.1**

a Here ‘a’ and ‘b’ represent T–T and G–G conformation of the initial geometry that was used for conformational analysis respectively.

**Fig. 4:**
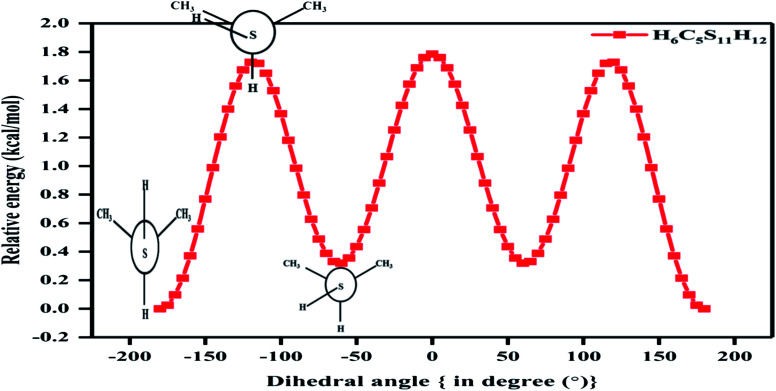
Potential energy surface plot of 2P molecule at CCSD/cc-pVDZ level of theory with respect to C–S bond (clockwise relaxed scan of step size 5°).

In the case of the 2-propanethiol (2P) molecule, a homologue of the nP molecule, no change in conformation was observed even after revisiting the calculations with higher levels of theory compared to the ones reported earlier. Similar to earlier reports,^12,13^ only two low-energy conformers were observed, with the anti conformer being the global minimum and the gauche conformer being the local minimum, as shown in Fig. 4 and SI1.† The potential energy surface corresponding to the HCSH dihedral angle with a step size of 5° from 0 to 360° was generated. The rotational barrier corresponding to the C–S bond was 1.72 kcal mol^−1^, closer to the experimentally measured value of 1.88 kcal mol^−1^.^13^ The energy difference between these conformers was calculated (single-point energy) to be 0.03 kcal mol^−1^ at the CCSD(T)/CBS limit as given in Tables 2 and ST5,† where the experimentally measured value was 0.06 kcal mol^−1^. The population analysis summarized in Table 3 reinforces anti conformer being the global minimum and gauche as the local minimum. The stability of conformers of 2P molecules was well correlated with the NCI plots, as shown in Fig. SI4(b).†

The thermodynamic parameters of conformers T–G and G–G of nP molecule and for conformers anti and gauche of 2P molecule are summarized in Table ST4.† Fausto *et al.*^11^ predicted 3.7 kcal mol^−1^ and cited Don Smith *et al.*'s experimental work of 1968 based on infrared absorption spectroscopy for the rotational barrier of 2P.^12^ However, Griffith *et al.* carried out microwave absorption spectroscopy in 1975, adding accuracy to the earlier observed conformers of 2P.^13^ Griffith *et al.* reported 1.88 kcal mol^−1^ as the rotational barrier, and it is beyond our comprehension as to why Fausto *et al.* cited Don Smith's work but ignored Griffith's. It must be noted that the values for the rational barrier predicted and reported in this work match exceedingly well with the values observed by Griffith *et al.*

### Electrostatics potential map (ESPM)

Electrostatic potential (ESP) surface analysis helps to understand the molecule's chemical reactivity.^19^ The computed ESP map of nP is shown in Fig. SI5(a),† and the corresponding charges are depicted in Table ST5.† From Table ST5,† it is clear that the sulfur atom (S_11_) behaves as an electrophilic center in all conformers. Specifically, the conformer T–T shows the highest values compared to T–G, G–G, and G–T. The electrophilicity of the C_1_ atom was seen to be the maximum in the T–T conformer and the third highest in the T–G conformer, whereas conformer G–G shows the least electrophilicity. The variation in the electrophilicity is the direct consequence of the variation in the extent to which the C_1_ atom interacts with the sulfur atom's lone pair of electrons. The major nucleophilic center is the C_5_ atom in T–T, T–G, and G–G conformers of the nP molecule. In the G–G conformer, the H_10_ atom strongly interacts with the lone pair of the sulfur atom, but atom H_9_ shows weak interactions compared to T–T conformers. ESP charge also confirms that the conformer G–G and T–G are the thermodynamically favorable conformers.

Similar to the nP molecule, the 2P molecule showed a similar ESP map as shown in Fig. SI5(b),† and the charges corresponding to each atom of 2P are summarized in Table ST5.† In the case of the 2P molecule, the sulfur atom (S_11_) is the most electrophilic center in the gauche conformer, whereas it is the least electrophilic in its anti-conformer. However, the carbon center (C_1_) is the most electrophilic in anti-conformer, and it becomes the second most electrophilic center in gauche-conformer. Similar to nP, in 2P also, the C_5_ atom is highly nucleophilic in both the conformers. Variation in nucleophilicity or electrophilicity center is mainly affected by the interaction of the electron-rich and electron-deficient centers of the molecules. NBO results validate these predictions very well, and the same are summarized below.

### Mulliken charge analysis (MCA)

MCA helps to analyze charge mobility and electronegativity processes within the molecule.^15^ Mulliken charges (MC) were computed for the T–G, T–T, G–G, and G–T conformers of nP and for conformers of 2P molecule, which are given in Table ST5.† Mulliken charges on the sulfur atom are negative for all conformers of the nP molecule. The values of the negative MC on the sulfur atom of T–G and G–T conformers are lower than in the T–T conformer, and hydrogen atoms 9 and 10 attain the maximum positive MC, indicating strong interactions that were well visualized in the NCI plot given in Fig. SI4.† In the case of the G–G conformer, the MC charge on the sulfur atom are positive and other atoms also have positive MC charges means atoms are weak in participation of interactions as compared to T–T and T–G conformer. NBO calculations also justified the delocalization of sulfur electrons into antibonding molecular orbital of the C–H_9/10_ bond, thereby making the T–G conformer more stable than the other two conformers of the nP molecule. These MCA calculations were further supported by the NBO calculations described below.

### NBO analysis

NBO calculations were carried out to know the extent of interaction (inter or intramolecular) between different orbitals (donor or acceptor orbital) of the nP and 2P and also understand the charge transfer phenomenon in terms of the second-order perturbation energy, *E*(2).^20^ The greater the *E*(2) values translate to greater interactions between each orbital and *vice versa*. In the NBO analysis, the chemical interpretation of hyper-conjugative interactions and transfer of electron density from the filled orbital or filled lone-pair electron occurs. NBO results for nP and 2P molecules are summarized in Tables ST6 and ST7.† It was found that the conformers G–G, T–T, and T–G become stable compared to the conformer G–T because the G–T conformer has fewer interactions than the G–G, T–T, and T–G conformers. The interaction of sulfur's lone pair of electrons with orbitals σ*C_8_–H_9_ and σ*C_8_–H_10_ takes place along with donation in a σ*C_5_–C_8_ orbital in G–G, T–T, and T–G conformers. The sulfur atom of T–T conformer shows strong interactions with σ*C_8_–H_9_ and σ*C_8_–H_10_ orbitals through both lone pairs, whereas T–G conformer only interacts with the σ*C_8_–H_9_ orbital because the σ*C_8_–H_10_ orbital is not in close proximity with no donation of the electrons to the antibonding molecular orbital of the C_8_–H_10_ bond. This means that the lone pair of the sulfur atom strongly interacts with hydrogen atoms 9 and 10 in the T–T conformer, whereas they interact only with the H_9_ hydrogen atom of the T–G conformer. This indicates that the sulfur atom has a significantly lower electron density in T–T than the T–G conformer rendering it the most robust electrophilic center in the T–T conformer. Moreover, the G–G conformer shows strong interaction between σ*C_5_–HC_8_ orbital and sulfur lone pair of electrons that gives maximum stabilization energy compared to other conformers of nP molecule. Furthermore, the sulfur atom of the G–G conformer interacts only with σ*C_8_–H_9_ orbital and not with σ*C_8_–H_10_ orbital. Based on these predictions, it is inferred that the Gibbs free energy (thermodynamically) favored conformers are G–G and T–G (thermodynamically) and the T–T conformer is favored by electrostatic interactions (*i.e.*, kinetically).

NBO analysis of the 2P molecule revealed 26 interactions in both the conformers (*i.e.*, gauche and anti). The most intense interaction occurs between the lone pair of the sulfur atom and the antibonding molecular orbitals (ABMO) of the C–C and C–H bonds. In the anti-conformer, the lone pair of the sulfur atom strongly interacts with ABMOs of the C_1_–C_5_ and C_5_–C_7_ bonds, but in the gauche conformer, this happens with ABMOs of the C_1_–C_5_ and C_5_–H_6_ bonds. The sulfur atom of the 2P molecule has almost similar positive values to the ESPs, meaning both conformers have the same electrophilic center capacity. Overall, *E*(2) values for the anti-conformer are more prominent than the gauche conformer. Consequently, the anti-conformer becomes the most stable conformer out of all possible conformers of the 2P molecule, and the gauche conformer is the second most stable conformer. The NBO analysis agrees with the ESP, MCA, FMO calculations for both nP and 2P molecules.

### FMO analysis

**Fig. 5:**
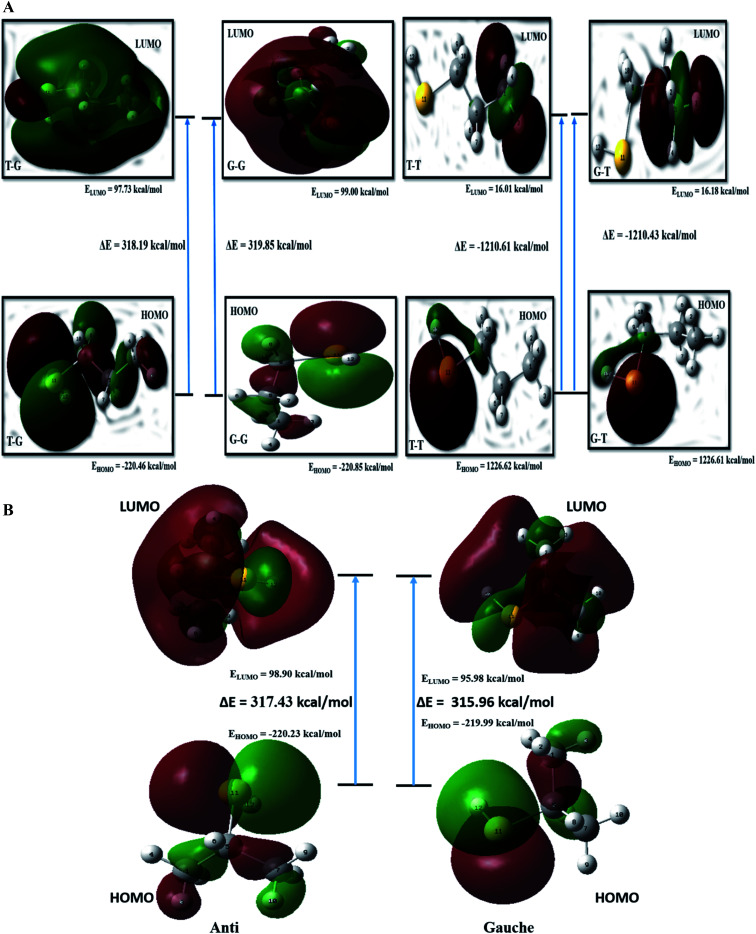
(a) Schematic representation of frontier molecular orbitals of the different conformers of nP molecule at CCSD/cc-pVDZ level of theory. (b) Schematic representation of frontier molecular orbitals of the different conformers of 2P molecule at CCSD/cc-pVDZ level of theory.

The kinetic stability depends on the HOMO–LUMO (H–L) gap.^20^ Values of H–L gaps define the chemical reactivity of a molecule. A lower value of the H–L gap indicates intense reactivity of the molecular system and *vice versa*. Energy parameters of the frontier molecular orbitals are summarized in Table ST8† and graphically represented in Fig. 5(a). The H–L gap for T–T and G–T conformers is significantly smaller, indicating relatively higher reactivity than the T–G and G–G conformers of the nP molecule. Hence T–G and G–G are inferred as the most stable conformers followed by the T–T conformer and G–T is the least stable conformer of the nP molecule. For the nP molecule, our calculated dipole moment for the T–G conformer perfectly agrees with the experimental value 1.60 D,^3^ as shown in Table ST8.† Out of the four conformers, G–G has maximum dipole moment (1.72 D), followed by the T–G conformer.

FMO calculation was also done for 2P molecule and given in Table ST8† and Fig. 5(b). The anti conformer has a more significant H–L gap than the gauche (315.96 kcal mol^−1^) conformer rendering it the more stable conformer than the gauche conformer. Theoretically calculated dipole moment for 2P molecule has a significantly closer value to the experimental value (1.61 D),^13^ and the same is given in Table ST8.† These predictions have excellent agreement with the ESP, MCA, and NBO analysis.

## Summary

The minimum energy conformer of nP and 2P was identified by rigorously exploring the conformation space pertaining to the dihedral angles. Adequate justification was given for the two conformers with the lowest energies. With the meticulous analysis and justification reported herein, the hitherto existing ambiguity in the computational literature is expected to cease. In summary,

• Four (T–G, G–G, T–T, and G–T) and two (anti and gauche) minimum energy conformers are found for nP and 2P molecules.

• Out of four conformers of nP molecule, T–G is the global minima with three local minima G–G, T–T, and G–T.

• In the conformational analysis of nP the role of the C–C and C–S bonds was seen to be highly crucial in determining the energy of the conformer.

• Conformational analysis of nP molecule with T–T conformer as the starting geometry matched well with the experimental values vis-à-vis the G–G conformer as the starting geometry.

• Conformational stability of nP and 2P molecules was corroborated through NBO, FMO, MCA, ESPM, and NCI analysis.

• Our calculated geometrical parameters and rotational barriers for nP and 2P molecules excellently matched the experimental results.

## Conflicts of interest

There are no conflicts to declare.

## Supplementary Material

RA-012-D2RA01034H-s001
