# Supplementary material for: Conformational and structural stability of n and 2-propylthiols: a revisit
Source: RSC Adv. 2022 Apr 1;12(17):10336–44. doi: 10.1039/d2ra01034h (PMC8973430; doi:10.1039/d2ra01034h)
Supplement: RA-012-D2RA01034H-s001 [file RA-012-D2RA01034H-s001.pdf]

Electronic Supplementary Material (ESI) for RSC Advances.
This journal is © The Royal Society of Chemistry 2022

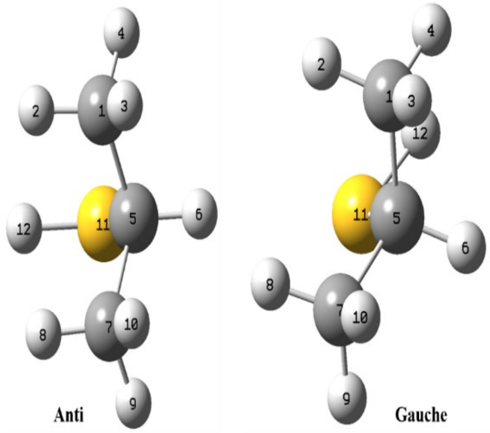

Fig. SI1. Optimized geometries of conformers of 2P molecule at CCSD/cc-pVDZ level of theory

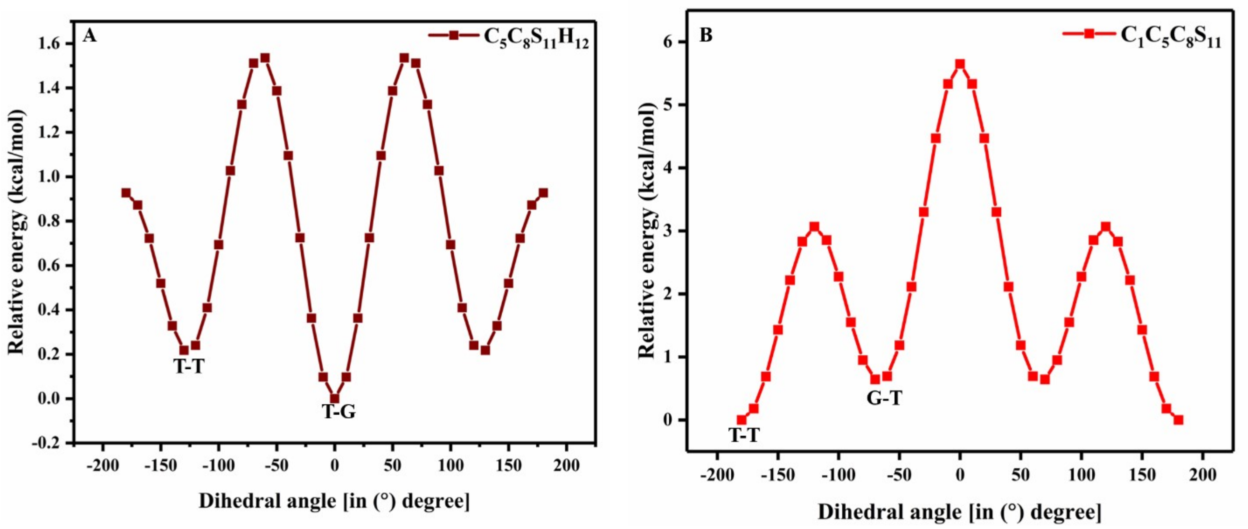

Fig. SI2 (a). Potential energy surface plots generated for nP molecule correspond to (A) C-S and (B) C-C bonds with step size 10º at B3LYP/6-311++G (2df 2pd) level of theory.

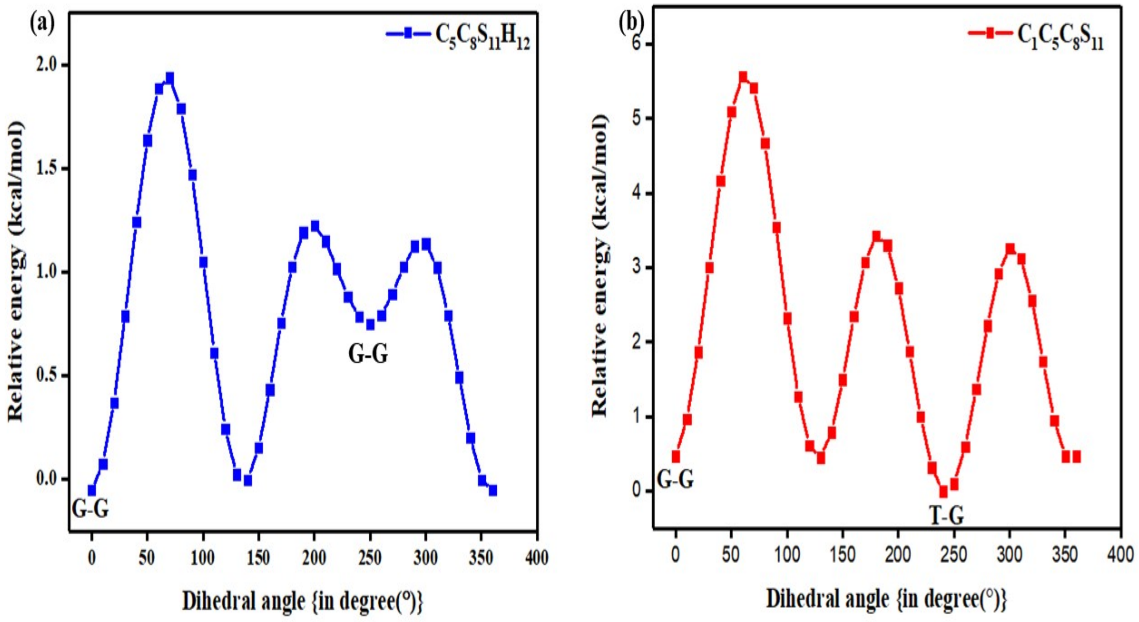

Fig. SI2 (b). Potential energy surface plots generated for nP molecule correspond to (a) C-S and (b) C-C bonds with step size 10º at CCSD/cc-pVDZ level of theory.

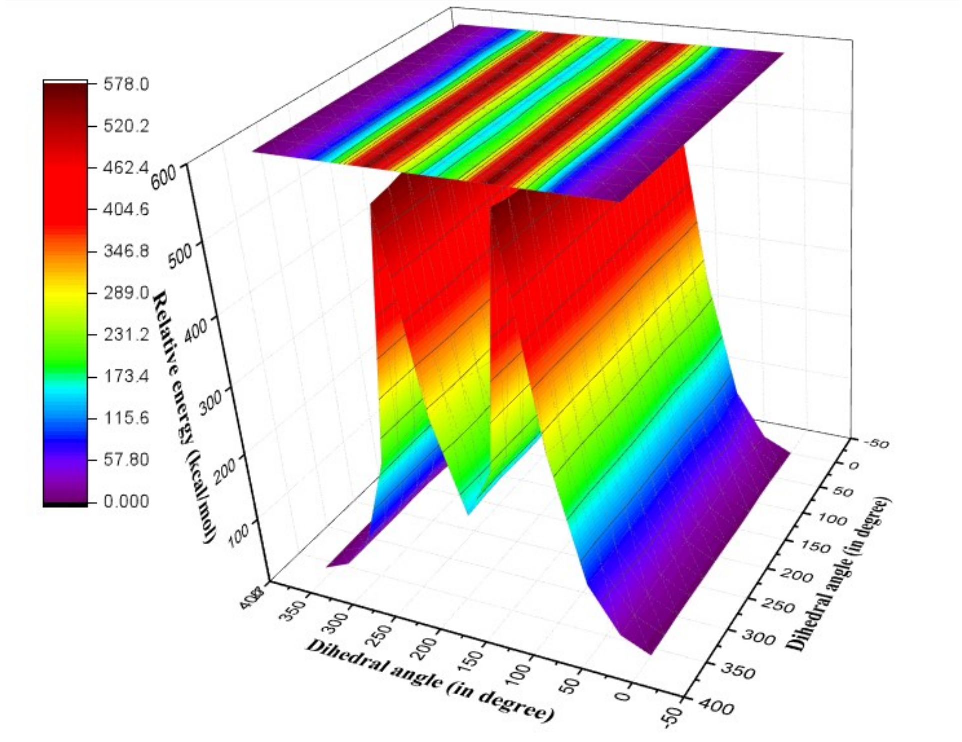

Fig. SI3 Potential energy surface plot generated for nP molecule with two coordinates (CCCS and CCSH) with step size 30º at CCSD/cc-pVDZ level of theory (geometry of G-G conformer as a starting molecule)

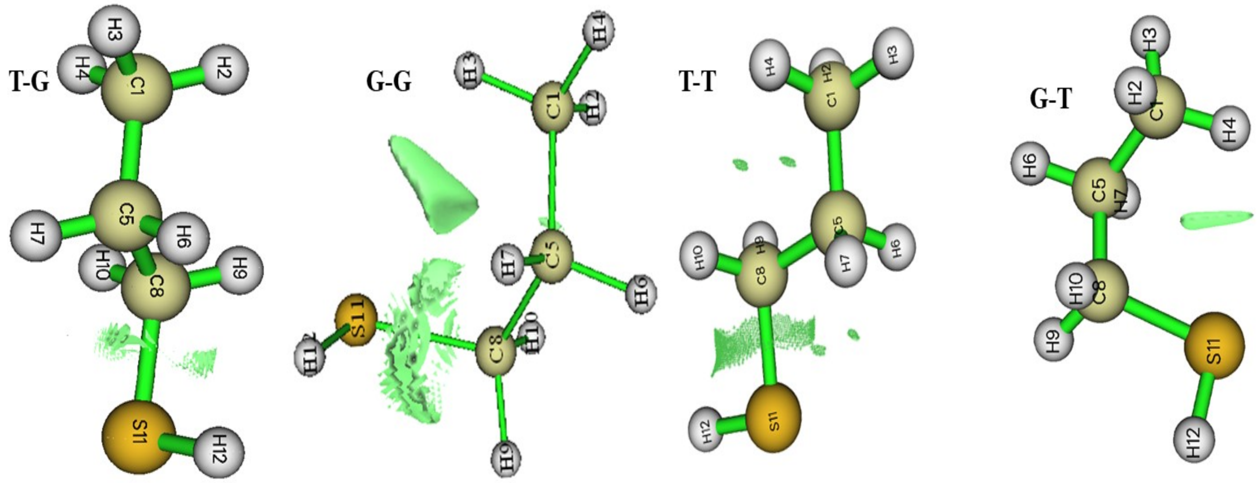

Fig SI4 (a). NCI Plot of conformers T-G, G-G, T-T, and G-T of nP molecule at CCSD/cc-pVDZ level of theory

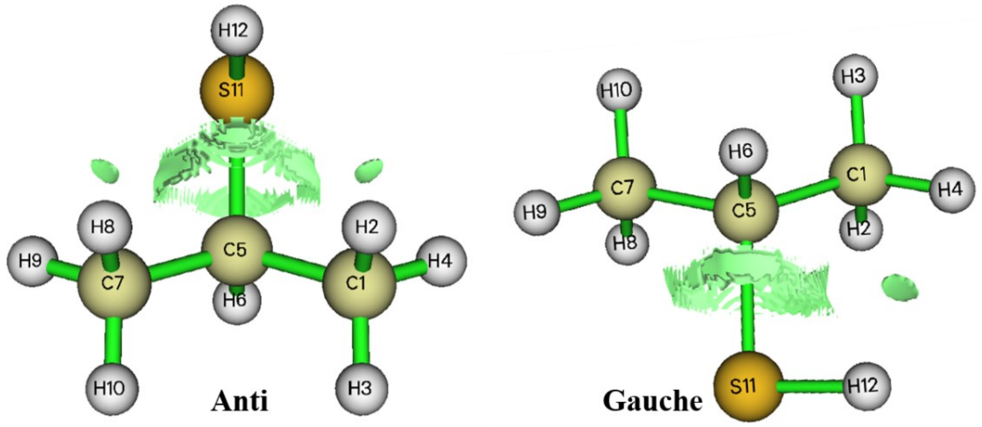

Fig. SI4 (b). NCI Plot of conformers anti, and gauche of 2P molecule at CCSD/cc-pVDZ level of theory

NCI plot

Non-covalent interaction plot gives a better way to visualize more precisely weaker interactions in molecules [1]. Herein the green color shows attractive non-covalent interaction between the donor and acceptor system. Conform G-G and T-T shows more number of interactions as compared to T-G and G-T conformers, which gives clues about the stability of the conformers. In the NCI plot of the T-T and G-G conformer, the sulfur atom interacts with C8 and C5 atoms and also the hydrogen atom of these carbon centers but this interaction is absent for the G-T conformer. Conformer T-G shows strong interactions with the hydrogen atom of the C_8 other than this no interaction was found. So the conformer with a higher number of interactions got the higher stability and they are referred to as kinetically favorable conformers. Thus, the conformer T-T is a kinetically favorable conformer, and the conformer T-G and G-G is the thermodynamically favorable conformer because it attains the lowest energy in possible conformers of the nP

molecule. NCI plot of 2P conformers also shows weak interactions between lone pair and the two adjacent carbon atoms in both anti and gauche conformers of 2P molecule. Gauche conformer shows one additional weak interaction because of the active participation of the sulfur's lone pair and antibonding molecular orbital of the C_1-H_4 bond. A similar kind of interaction is also found in the anti-conformer of the 2P molecule with one additional interaction that was formed between the sulfur lone pair and the ABMO of the C_7-H_9 bond. NCI plots of the weak interactions reinforced very well with results of the MCA, ESP, FMO, and NBO analysis.

Reference:

1. Monika Pareek, Raghavan B Sunoj, Mechanism and steresoselectivity in an Asymmertic N-Heterocyclic carbine-catalyzed carbon-carbon bond activation reaction, Organic letters, 18, 2016, 5932-5935.

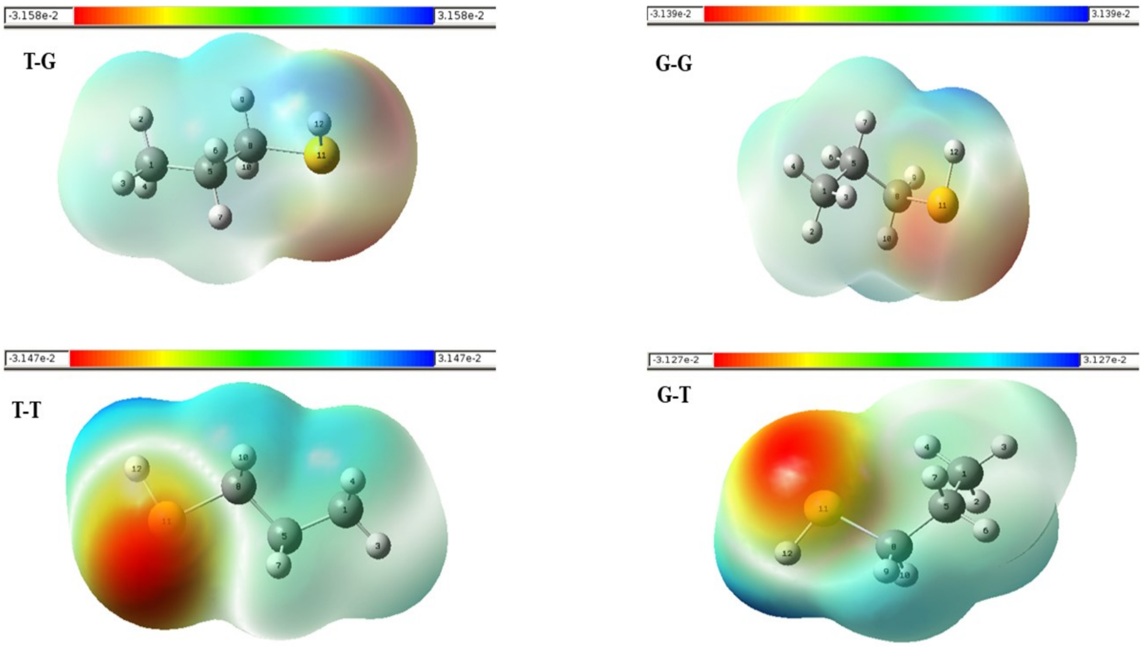

Fig.SI5 (a). Electrostatic potential (ESP) surface plot of conformers of nP molecule at CCSD/cc-pVDZ level of theory.

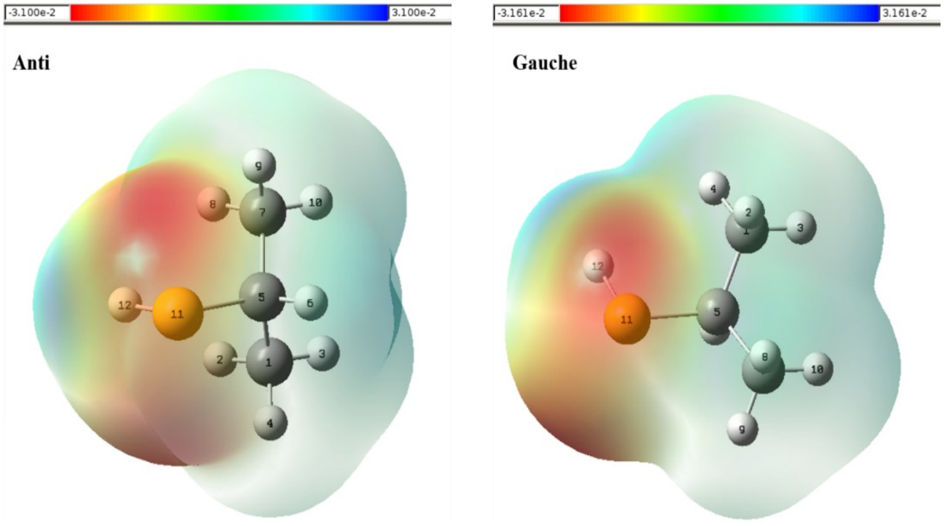

Fig. SI5 (b). Electrostatic potential (ESP) surface plot of conformers of 2P at CCSD/cc-pVDZ level of theory.

Table: ST1 Fundamental vibrational [Infrared (IR)] frequencies (cm^{-1}) of the conformers of nP and 2P molecules at CCSD/cc-pVDZ level of theory

n-propanethiol (nP)		
IR [T-T]	IR [T-G]	Experimental (IR)[1-3]
183 [τ_{S-H}]	211 [τ_{S-H}]	178
357 [δ_{C-C-C}]	367 [δ_{C-C-C}]	329
753 [ν_{C-S}]	715 [ν_{C-S}]	700
756 [ρ_{CH2}]	748 [ρ_{CH2}]	728
872 [ρ_{CH2}]	815 [δ_{S-H}]	814
1147 [mixed ν_{C-C} & ω_{CH2}]	1143 [mixed ν_{C-C} & ω_{CH2}]	1105 [ν_{C-C}]
1252 [τ_{CH2}]	1258 [τ_{CH2}]	1243
1275 [ω_{CH2}]	1280 [ω_{CH2}]	1300
1390 [ω_{CH2}]	1384 [ω_{CH2}]	1351
1423 [δ_{CH3}]	1421 [δ_{CH3}]	1384
1491 [ρ_{CH2}]	1477 [ρ_{CH2}]	1456
2731 [ν_{S-H}]	2725 [ν_{S-H}]	2598
3055 [$\nu_{CH3(s)}$]	3054 [$\nu_{CH3(s)}$]	2838
3063 [$\nu_{CH2(s)}$]	3062 [$\nu_{CH2(s)}$]	2848
3076 [$\nu_{CH2(s)}$]	3077 [$\nu_{CH2(s)}$]	2945
3102 [$\nu_{CH2(a)}$]	3105 [$\nu_{CH2(a)}$]	2960
3130 [$\nu_{CH2(a)}$]	3129 [$\nu_{CH2(a)}$]	3090
3142 [$\nu_{CH3(a)}$]	3142[$\nu_{CH3(a)}$]	3183
2-propanethiol (2P)		
IR (Anti)	IR (Gauche)	Experimental (IR)[1-3]
211 [τ_{S-H}]	197 [τ_{S-H}]	185
250 [τ_{CH3}]	253 [τ_{CH3}]	245
277 [τ_{CH3}]	273 [τ_{CH3}]	310
332 [δ_{C-C-S}]	306 [δ_{C-C-S}]	325
409 [δ_{C-C-C}]	414 [δ_{C-C-C}]	410
635 [ν_{C-S}]	652 [ν_{C-S}]	620
873 [δ_{S-H}]	875 [δ_{S-H}]	853
934 [ρ_{CH3}]	938 [ρ_{CH3}]	955
967 [ρ_{CH3}]	1085 [ρ_{CH3}]	1063
1118 [ρ_{C-C}]	1172 [ν_{C-C}]	1082
1312 [δ_{C-H}]	1364 [ω_{C-H}]	1314
1348 [ω_{C-H}]	1413 [ω_{CH3}]	1389
1410 [ω_{CH3}]	1423 [ω_{CH3}]	1448
2716 [ν_{S-H}]	2727 [ν_{S-H}]	2572
3050 [$\nu_{CH3(s)}$]	3051 [$\nu_{CH3(s)}$]	2927

Note: Tentative assignment of vibrational mode given inside the parenthesis

Reference:

1. Torgrim Torgrimsen, Peter Klaeboe, The vibrational spectra and the stable conformers of 1-propanethiol, Acta Chem. Scand, 24, 1970.
2. Don Smith, J Paul Devlin, Conformational analysis of ethanethiol and 2-propanethiol, Journal of Molecular Spectroscopy, 25, 1968, 174-184.
3. Sunyoung Choi, Tae Yeon Kang, Kyo-Won Choi, Songhee Han, Doo-Sik Ahn, Sun Jong Baek, Sang Kyu Kim, Conformationally specific vacuum ultraviolet mass-analyzed threshold ionization Spectroscopy of alkanethiols: Structure and ionization of conformational isomers of ethanethiol, isopropanethiol, 1-propanethiol, tert-butanethiol, and 1-Butanethiol, The Journal of Physical Chemistry A, 112, 2008, 7191-7199.

Table: ST2 Summary of conformational analysis of nP molecule via relaxed scan at CCSD/cc-pVDZ level of theory.

Scan coordinate	Global minimum	Local minimum	Rotational barrier (kcal/mol)	Relative energy change (kcal/mol)	Experiment (kcal/mol)
$C_1C_5C_8S_{11}$	T-T	G-T	3.14 (C-C)	0.37	2.9[2]
$C_5C_8S_{11}H^a_{12}$	T-G	T-T	1.58 (C-S)	0.72	1.31[1]
$H_6C_5C_8H_9$	T-T	G-T	3.13 (C-C)	0.36	2.9[2]
$H_6C_5C_8S_{11}$	T-T	G-T	3.10 (C-C)	0.38	2.9[2]
$C_1C_5C_8S_{11}$	T-G	G-G	3.93 (C-C)	0.22	2.9[2]
$C_5C_8S_{11}H^a_{12}$	G-G	G-G’	2.24 (C-S)	0.12	1.31[1]

Note: [a]optimized geometry of T-T conformer; [b]optimized geometry of G-G conformer, have been taken into consideration.

Reference:

1. Jun Nakagawa, Michiro Hayashi, Internal rotation in propyl mercaptan by microwave spectroscopy, J. of Mol. Spect., 85 (1981), pp. 327-340.
2. R. E. Pennington, D. W. Scott, H. L. Finke, J. P. McCullough, J. F. Messerly, I. A. Hossenlopp, Guy Waddington., The Chemical Thermodynamic Properties and Rotational Tautomerism of 1-Propanethiol1, J. of the Amer. Chem. Soci., 78 (1956), pp. 3266-3272.

Table: ST3 Relative change in energy (kcal/mol) of the local minima conformers of the nP and 2P molecules with respect to the global minima conformer at a different level of theory.

Basis set→	cc-pVDZ		cc-pVTZ		cc-pVQZ		CBS limit		Experiment	
Methods↓	nP	2P	nP	2P	nP	2P	nP	2P	nP	2P
HF	0.48	0.14	0.68	0.04	0.75	0.06	0.78	0.070	0.38[1]	0.06[2]
MP2	0.01	0.35	0.11	0.10	0.13	0.04	0.16	0.004		
MP3	0.18	0.36	0.25	0.10	0.28	0.06	0.30	0.031		
MP4	0.17	0.36	0.22	0.10	0.26	0.06	0.28	0.031		
CCSD	0.19	0.32	0.25	0.12	0.28	0.03	0.30	0.032		
CCSD(T)	0.14	0.37	0.17	0.11	0.20	0.06	0.22	0.030		

Reference:

1. Jun Nakagawa, Michiro Hayashi, Internal rotation in propyl mercaptan by microwave spectroscopy, J. of Mol. Spect., 85 (1981), pp. 327-340.
2. John H. Griffiths, James E. Boggs, Microwave spectrum and rotational isomerism in isopropyl mercaptan, J. of Mol. Spect., 56 (1975), pp. 257-269.

Table: ST4 Thermodynamic parameters of conformers of nP and 2P molecules at CCSD/cc-pVDZ level of theory (all parameters are in kJ/mol)

Thermodynamic Parameters	n-propanethiol (nP)			2-propanethiol (2P)	
	T-G	G-G	T-T	Anti	Gauche
Gibbs Free energy (ΔG) kJ/mol	-1355705.43	-1355704.30	-1355702.86	-1355711.50	-1355710.33
Enthalpy (ΔH) kJ/mol	-1355611.44	-1355610.64	-1355608.18	-1355618.83	-1355617.40
Entropy (ΔS) kJ/mol	0.32	0.31	0.32	0.31	0.31
Heat capacity (Cv) kJ/mol	0.08	0.08	0.08	0.09	0.09

Thermodynamic parameters also help in the elucidation of the stability of molecules [1]. So for this, we computed thermodynamic parameters at our benchmark functional CCSD/cc-pVDZ because results of this functional exceedingly matched with experimental results, which is summarized in Table ST7. From Table ST7, conformer T-G is the global minimum conformer for nP molecule and conformer anti is the global minimum for 2P molecule.

Thermodynamic results reinforced predictions of FMO analysis where conformer T-G is the thermodynamically favorable conformer of nP molecule and ant conformer is the kinetically and thermodynamically favorable conformer for 2P molecule.

Reference:

1. E. M. Cabaleiro-Lago and J. Rodríguez-Otero, Methanethiol dimer and trimer. An ab initio and DFT study of the interaction, J. Phys. Chem. A, 2002, 106(32), 7440–7447

Table: ST5. Absolute values of electrostatic potential charge (ESP) and mulliken charges (MC) on the atom of the conformers of nP and 2P molecules at CCSD/cc-pVDZ level of theory (All values are expressed in atomic units and parenthesis contains mulliken charge)

Atom label	nP				2P	
	T-T	G-G	T-G	G-T	Gauche	Anti
1 C of CH_3	-0.424 (-0.061)	-0.176 (0.059)	-0.223 (-0.062)	-0.203 (-0.066)	-0.371 (0.002)	-0.503 (0.004)
2 H of CH_3	0.108 (0.036)	0.055 (0.030)	0.047 (0.034)	0.042 (0.035)	0.093 (0.046)	0.146 (0.032)
3 H of CH_3	0.091 (0.040)	0.036 (0.054)	0.055 (0.041)	0.035 (0.037)	0.113 (0.036)	0.135 (0.037)
4 H of CH_3	0.108 (0.036)	0.036 (0.036)	0.032 (0.036)	0.081 (0.050)	0.098 (0.035)	0.114 (0.050)
5 C of CH_2-CH_3	0.259 (0.093)	0.134 (0.079)	0.272 (-0.073)	0.279 (-0.094)	0.216 (-0.281)	0.277 (-0.286)
6 H of CH_2-CH_3	0.021 (0.044)	-0.023 (0.035)	-0.048 (0.032)	-0.043 (0.037)	0.032 (0.063)	0.120 (0.068)
7 H of CH_2-CH_3	0.021 (0.044)	0.005 (0.031)	-0.057 (0.045)	-0.005 (0.042)	-0.275 (-0.018)	-0.503 (0.004)
8 C of CH_2-SH	-0.297 (-0.156)	-0.098 (0.150)	-0.089 (-0.162)	-0.284 (-0.143)	0.120 (0.047)	0.146 (0.032)
9 H of CH_2-SH	0.127 (0.060)	0.041 (0.070)	0.029 (0.068)	0.110 (0.061)	0.092 (0.052)	0.114 (0.050)
10 H of CH_2-SH	0.127 (0.060)	0.129 (0.072)	0.124 (0.072)	0.125 (0.059)	0.068 (0.042)	0.135 (0.037)
11 S	-0.345 (-0.075)	-0.329 (0.107)	-0.321 (-0.099)	-338 (-0.084)	-0.376 (-0.092)	-0.369 (-0.093)
12 H of S	0.204 (0.064)	0.188 (0.069)	0.178 (0.068)	0.200 (0.066)	0.190 (0.068)	0.189 (0.065)

Table: ST6. Summary of NBO analysis of conformers of nP molecule at CCSD/cc-pVDZ level of theory

Orbital Interaction	Second order perturbation energy [E(2)] (kcal/mol)			
	T-T	G-G	G-T	T-G
σ1C-H2→ σ*5C-H7	4.32	4.57	4.46	4.32
σ1C-H3→ σ*5C-H6		4.53		
σ1C-H3→ σ*5C-H8	4.89		5.20	4.88
σ1C-H4→ σ*5C-H6	4.32		4.23	4.37
σ1C-H4→ σ*5C-C8		5.19		
σ1C-H5→ σ*5C-C8		0.76	0.71	0.82
σ1C-H5→ σ*5C-H6			2.39	
σ1C-H5→ σ*8C-H9		2.46		
σ1C-H5→ σ*8C-S11	3.74			4.30
σ5C-H6→ σ*1C-H4			4.62	4.55
σ5C-H6→ σ*1C-H3		4.56		
σ5C-H6→ σ*1C-H4	4.54			
σ5C-H6→ σ*8C-H10	4.58			4.68
σ5C-H6→ σ*8C-S11		7.13	6.44	
σ5C-H7→ σ*1C-H2	4.54	4.48	4.49	4.54
σ5C-H7→ σ*8C-H9	4.58			4.70
σ5C-H7→ σ*8C-H10		4.62	4.54	
σ5C-C8→ σ*1C-H3	2.40		2.32	2.34
σ5C-C8→ σ*1C-H4		2.34		
σ5C-C8→ σ*1C-C5		0.86	0.78	0.78
σ5C-C8→ σ*8C-H9		0.51	0.57	
σ5C-C8→ σ*8C-H10		0.53		0.56
σ5C-C8→ σ*11S-H12			0.70	
σ8C-H9→ σ*1C-C5		4.65	4.81	
σ8C-H9→ σ*5C-H7	4.36		4.34	4.21
σ8C-H10→ σ*5C-H6	4.36			4.39
σ8C-H10→ σ*5C-H7		4.36	4.34	
σ8C-H10→ σ*11S-H12		1.98		1.99
σ8C-S11→ σ*5C-H6		3.00	3.46	
σ8C-S11→ σ*1C-C5	4.17			3.61
σ8C-S11→ σ*11S-H12	0.59	0.67	0.56	0.65
σ8C-H9→ σ*5C-H6	4.36			
σ8C-H10→ σ*5C-H6	4.36			
σ11S-H12→ σ*5C-C8	2.75		2.96	
σ11S-H12→ σ*8C-S11	1.01	1.15	0.92	1.19
σ11S-H12→ σ*8C-H10		2.90		2.96
LP(1)11S→ σ*5C-C8		1.29		1.26
LP(1)11S→ σ*1C-C5	0.51			
LP(1) 11S→ σ*8C-H9	1.14	1.37	1.11	1.41
LP(1)11S→ σ*8C-H10	1.14		0.99	
LP(2)11S→ σ*8C-H9	5.28	4.30	4.60	4.91
LP(2)11S→ σ*8C-H10	5.28		5.57	

LP(2)11S→ σ*1C-C5				1.13
LP(2)11S→ σ*1C-H3		0.60		
LP(2)11S→ σ*5C-C8		5.64		5.10
LP(2)11S→ σ*5C-H6		0.94		

Table: ST7. Summary of NBO analysis of conformers of the 2P molecule at CCSD/cc-pVDZ level of theory

Interacting orbitals		Gauche			Anti		
Donor	Acceptor	E(2) (kcal/mol)	$E_{(j)}$-$E_{(i)}$ (atomic unit)	F (i, j) (atomic unit)	E(2) (kcal/mol)	$E_{(j)}$-$E_{(i)}$ (atomic unit)	F (i, j) (atomic unit)
C1 - H2	C5 - H6	4.48	1.39	0.071	4.52	1.38	0.071
C1 - H3	C5 - S11	6.59	1.03	0.074	6.50	1.04	0.073
C1 - H4	C5 - C7	4.78	1.30	0.070	4.79	1.30	0.071
C1 - C5	C5 - H6	0.51	1.54	0.025	0.66	1.53	0.029
C1 - C5	C5 - C7	1.15	1.45	0.037	0.99	1.45	0.034
C1 - C5	C7 - H9	2.59	1.53	0.056	2.52	1.54	0.056
C5 - H6	C1 - H2	4.39	1.38	0.070	4.49	1.37	0.070
C5 - H6	C7 - H8	4.49	1.38	0.070	4.49	1.37	0.070
C5 - H6	S11 - H12				2.10	1.12	0.043
C5 - C7	C1 - H4	2.65	1.52	0.057	2.52	1.54	0.056
C5 - C7	C1 - C5	1.06	1.45	0.035	0.99	1.45	0.034
C5 - C7	C5 - H6	0.57	1.54	0.027	0.66	1.53	0.029
C5 - C7	S11 - H12	0.87	1.27	0.030			
C5 - S11	C1 - H3	3.55	1.45	0.064	3.54	1.46	0.064
C5 - S11	C7 - H10	4.02	1.46	0.068	3.54	1.46	0.064
C5 - S11	S11 - H12	0.69	1.20	0.026	0.76	1.20	0.027
C7 - H8	C5 - H6	4.38	1.39	0.070	4.52	1.38	0.071
C7 - H9	C1 - C5	4.73	1.30	0.070	4.79	1.30	0.071
C7 - H10	C5 - S11	5.77	1.04	0.069	6.50	1.04	0.073
S11 - H12	C5 - C6				2.84	1.42	0.057
S11 - H12	C5 - C7	2.77	1.33	0.054			
S11 - H12	C5 - S11	1.08	1.07	0.030	1.24	1.08	0.033
LP(1) S11	C1 - C5	1.16	1.42	0.036	1.47	1.42	0.041
LP(1) S11	C1 - C7				1.47	1.42	0.041
LP(1) S11	C5 - H6	1.23	1.51	0.039			
LP(1) S11	C7 - H10	0.52	1.49	0.025			
LP(2) S11	C1 - H3	1.25	1.05	0.033	1.30	1.05	0.033
LP(2) S11	C1 - C5	4.85	0.99	0.062	4.55	0.99	0.060
LP(2) S11	C5 – C7				4.55	0.99	0.060
LP(2) S11	C5 - H6	4.86	1.08	0.065			
LP(2) S11	C7 - H10				1.30	1.05	0.033

Table: ST8. Calculated energy parameters of conformers of nP and 2P molecules at CCSD/cc-pVDZ level of theory. (values in parenthesis shows experimental values)

Energy Parameter ↓	nP				2P	
	T-G	G-G	T-T	G-T	Anti	Gauche
E_{HOMO} (IP) (kcal/mol)	-220.46	-220.85	1226.62	1226.61	-220.23	-219.99
E_{LUMO} (EA) (kcal/mol)	97.73	99.00	16.01	16.18	98.90	95.98
LUMO - HOMO gap (E_g)	318.19	319.85	-1210.61	-1210.43	317.43	315.96
Dipole moment (D)	1.58 (1.60)[1]	1.72	1.51	1.48	1.766 (1.61)[2]	1.770
Hardness (η)	159.10	159.73	-605.31	-605.22	158.72	157.98
Chemical Potential (μ)	-61.37	-60.73	621.32	621.40	-60.67	-62.01
Electronegativity (χ)	61.37	60.73	-621.32	-621.40	60.67	62.01
Elecrophilicity Index (ω)	11.84	11.54	-318.88	-319.01	11.60	12.17

[1] Jun Nakagawa, Michiro Hayashi, Internal rotation in propyl mercaptan by microwave spectroscopy, J. of Mol. Spect., 85 (1981), pp. 327-340.

[2] John H. Griffiths, James E. Boggs, Microwave spectrum and rotational isomerism in isopropyl mercaptan, J. of Mol. Spect., 56 (1975), pp. 257-269.
